# Rapid Campimetry in glaucoma – correspondence with standard perimetry and OCT

**DOI:** 10.1038/s41598-024-75037-5

**Published:** 2024-10-25

**Authors:** Nidele Djouoma, Fabian Müller, Francie H. Stolle, Friedrich Hoffmann, Hagen Thieme, Michael B. Hoffmann, Khaldoon O. Al-Nosairy

**Affiliations:** 1 Ophthalmic Department, Faculty of Medicine, Otto-von-Guericke University, Leipziger Str. 44, 39120 Magdeburg, Germany; 2 H & M Medical Solutions GmbH, 14195 Berlin, Germany; 3 Ophthalmology Department, Charité—Universitätsmedizin Berlin, 12203 Berlin, Germany; 4 Center for Behavioral Brain Sciences, Magdeburg, Germany

**Keywords:** Rapid Campimetry, VF, Kinetic VF, OCT, Glaucoma, Structure-function correlation, Arcuate scotoma, Telemedicine, Neuroscience, Visual system, Motion detection, Retina

## Abstract

The Rapid Campimetry (RC), a kinetic visual field test proved to reliably detect visual field defects within the central 10° degrees, the most crucial part for visual acuity and quality of life, affected even at very early stages of glaucoma, within a short measurement epoch, ~ 1 min. This study aims to further investigate RC correspondence with standard tests in glaucoma, namely standard automated perimetry (SAP) and optical coherence tomography (OCT) within 10° of visual field (VF). For this purpose, we included 41 participants, [21 glaucoma (GLA, mean age: 65.9 ± 12.4; 12 preperimetric eyes and 11 with VF defects) and 20 healthy controls (HC; mean age: 65.0 ± 10.3); 20 eyes]. At first, we compared the rate of detection/exclusion of VF defects in RC vs. SAP. Then, for those with VF defects (11 eyes), we investigated the 68-pointwise correspondence of 10 − 2 layout of RC and SAP. For functional and structural (SF) correspondence, pointwise correspondence of RC, SAP vs. OCT_macula_ [ macular ganglion cell thickness (GCL)] was also performed. Further, we compared sector-based correspondence of RC, SAP vs. OCT_macula_ accounting for GCL displacement at the fovea as well as sector-based correspondence with OCT_disc_ [peripapillary retinal nerve fiber layer thickness (pRNFL)]. Agreement estimates were reported along with Cohen Kappa ($\:\kappa\:$) statistic. For overall performance, RC and SAP showed 100% agreement ($\:\kappa\:=1$) for the exclusion of VF defects (HC and preperimetric GLA) and for detection of VF defects [11 eyes of 9 GLA, ($\:\kappa\:=1)$]. Further, RC outperformed SAP in detection of arcuate scotomas, 7 vs. 5, respectively. Pointwise correspondence of VF defects (11 eyes), RC-SAP agreement reached 90% accuracy ($\:\kappa\:=0.77,\:substantial\:agreement$). For SF correspondence, RC [SAP] showed 62% [69%] pointwise agreement with OCT_macula_, ($\:\kappa\:=0.10\:\left[0.33\right]$). For macular sector-based correspondence, SF correspondence improved and reached 83% [83%] agreement, ($\:\kappa\:=0.57\:\left[0.57\right]$). For OCT_disc_ sector-based analysis, SF correspondence was highest, 100% [100%] agreement, ($\:\kappa\:=1\:\left[1\right]$). Rapid Campimetry gave reproducible results in comparison to SAP and OCT with high potential as a screening VF method given its short testing duration, ~ 1 min in screening mode, and compatibility with telemedicine technologies upon future optimization and automation.

**Supplementary Information:**

The online version contains supplementary material available at 10.1038/s41598-024-75037-5.

## Introduction

A long unresolved yet pressing question in glaucoma practice is, whether damage in the macula, i.e. the most relevant region associated with individuals’ quality of life, exists already at early glaucomatous stages. The answer to this question has great impact on glaucoma management and importantly the life of the affected individuals. More than a century ago, Von Graefe^1^ was the first to describe paracentral scotoma in the central visual field (VF) of glaucoma cases. Traquair^2^ then introduced the concept of central VF damage as the earliest event in glaucoma damage, findings corroborated afterwards by Aulhorn^3^ and Drance^4,5^. Eventually, a number of recent reports reinforce the importance of the macular region^6–8^, as detailed below. As an alternative, the viewpoint can be taken that glaucoma is predominantly a peripheral disease and that the central VF is affected only later in advanced glaucomatous damage. Fortunately, contemporary endeavours to discern glaucoma damage, a common global cause of irreversible blindness affecting retinal ganglion cells, have re-addressed this issue and have provided compelling evidence of central VF involvement in early glaucoma.

Early macular glaucomatous damage was particularly evident by adopting tests other than common practice, i.e., standard automated perimetry (SAP) 24 − 2 VF test. Poor sampling of the 24 − 2 VF test with 6° degree spacing with only 8 central test points was reported to be insufficient to probe the macular region populated with ~ 50% of retinal ganglion cells^9^. Some investigators, hence, favor 10 − 2 SAP test, 2° spacing and 68 central points, to investigate macular damage at early stages of glaucoma. De Mores et al.^7^ found that 37% of glaucoma suspects, 34% of ocular hypertensives and 61% of early glaucoma have abnormal 10 − 2 VF in contrast to normal corresponding regions of the 24 − 2 VF test. In line with these findings, Traynis et al.^8^ reported abnormal 10 − 2 VF in 22% of early glaucoma eyes which had normal 24 − 2 VF. Structural evidence using macular optical coherence tomography (OCT) is also well documented in early glaucoma as shown by Hood et al., see review^6^. Even a baseline 10 − 2 VF is of help in glaucoma management by successfully predicting 24 − 2 VF progression, a report of a longitudinal study of ~ 7 years for over 300 eyes^10^.

Recently, a study addressed the utility of 10 − 2 VF in early glaucoma^11^ and reviewed the literature where 21 studies were included and summarized. Authors concluded that 10 − 2 VF is to be recommended in early glaucoma eyes with abnormal central 24 − 2 VFs regions and abnormal inner retinal thinning shown in OCT. Authors added that the routine use of 10 − 2 VF in early glaucoma must be weighed against potential costs, time, resources burdening patients and health system. In fact, the application of the 10 − 2 VF test is a more common strategy in advanced glaucoma^11^, which represents around one third of the presented stage in glaucoma^12^. However, between-visit variability and the limited dynamic range of VF testing in advanced glaucoma are limiting factors. Further, advanced glaucoma patients are more prone to being functionally impaired which imposes more costs on the health system^13,14^. Hence, given the importance of the central VF-region in patients across glaucoma continuum, both early and advanced, and its associated with quality of life and daily activities of glaucoma individuals^15–17^, further tests are warranted to fill this gap. Such tests should be less time consuming, of less burden on health system, and more accessible.

A recent kinetic visual field test termed Rapid Campimetry (RC) has been developed for glaucoma assessment in the central 10° region with the potential to outweigh disadvantages of the routine use of conventional 10 − 2 tests^18^. In fact, RC offers few advantages over a conventional standard automated perimetry (SAP = VF test: i) less time consuming, < 1 min; ii) suprathreshold stimulus suitable for VF screening and iii) potential for cloud telemedical technologies upon further optimization/automation. In our previous study^18^, we showed that RC is qualitatively comparable to standard 10 − 2 VF tests and has better performance in the detection of absolute arcuate scotoma. Further, we have also provided evidence^19^ that RC is robust to home-like suboptimal testing environment, refractive error, media opacity or ambient light, with good test-retest reproducibility in the same or different days. Motivated by these initial findings, further validation of RC in comparison to SAP and OCT, both in early and advanced glaucoma cases, is yet warranted for future implementation as a screening test for central 10° in glaucoma.

Thus, this study aims to compare RC vs. 10 − 2 SAP VF with respect to: (i) rate of VF defects detection within ± 10° of VF, (ii) detection of absolute arcuate scotoma, one of the initial and most common scotomas in glaucoma; and estimate the correspondence of RC vs. SAP and OCT via (iii) pointwise correspondence for 68 grid points of 10 − 2 topographies of each test and, (iv) structure function (SF) correspondence accounting for ganglion cell layer (GCL) displacement at the fovea using macular deviation maps, OCT_macula_, and (v) SF correspondence with OCT_disc_ estimates, namely peripapillary retinal nerve fiber layer (pRNFL) thickness.

## Methods

This cross-sectional study was performed in accordance with the tenets of the declaration of Helsinki and approved by the local ethics committee at the department of ophthalmology (no. 151/16), Otto-von-Guericke university, Magdeburg, Germany. All participants gave their written informed consent.

### Participants

In this study, 21 glaucoma patients (mean ± SD: 65.9 ± 12.4) were included, comprising 11 eyes from 9 perimetric glaucoma (GLA) and 12 eyes from 12 preperimetric glaucoma (GLA), along with 20 healthy controls (HC) contributing 20 eyes (65.0 ± 10.3 years) according to the inclusion and exclusion criteria (see below). All participants underwent a detailed ophthalmic examination and refractive correction for best-corrected visual acuity (BCVA). Visual function and structure tests were also performed successively as described below starting with RC, followed by SITA 10 − 2 visual field test (VF), and finally optical coherence tomography (OCT). For HC, both eyes were measured, but only one eye was randomly included in the analysis. According to common practice^7^, for GLA, if both exhibited glaucomatous visual field (VF) defects, both eyes were included in the analysis; if only one eye had VF defects, that eye was included, or if neither eye had defects, one eye was randomly selected. Table 1 shows an overview of participant characteristics.

### Inclusion and exclusion criteria

All participants had best corrected visual acuity (BCVA) > 0.8, except if related to glaucomatous damage. Participants with spherical equivalent refractive error ≤ ± 5 D and astigmatism < 2 D were included. Participants with any ocular, neurological or systemic disease affecting visual function other than early cataract were excluded. For both HC and GLA complete eye exam including slit-lamp, refractive correction, VF test and retinal integrity test with optical coherence tomography (OCT) was conducted. HC had normal ocular function and examination results, see Table 1.

Preperimetric open angle GLA was defined^20^ as participants having open anterior chamber angle with glaucomatous optic disc damage, retinal nerve fiber layer defect and/or a vertical cup disc ratio ≥ 0.7. Perimetric GLA had an additional visual field defects meeting Hodapp-Parrish-Anderson criteria^21^. Severity of VF defects was also defined after Hodapp criteria^21^.

**Table 1:** Participants characteristics.

Variable	N	Groups			ANOVA	Post-hoc		
		Control *n* = 20 of 20 HC^1^	Preperimetric Glaucoma *n* = 12 eyes of 12 GLA ^2^	Glaucoma *n* = 11 eyes of 9 GLA^3^	P-Value	1 vs. 2	1 vs. 3	2 vs. 3
Sex (m, f)	20,21	8, 12	6, 6	6, 3	0.431			
		Mean ± SD						
Age [Years]	41	65.0 ± 10.3	62.2 ± 13.8	70.9 ± 8.4	0.209			
BCVA [logMAR]	41	0.1 ± 0.1	-0.02 ± 0.1	0.1 ± 0.2	< 0.001	0.333	< 0.001	0.055
MD [dB]	41	0.3 ± 1.2	-0.5 ± 1.2	-16.4 ± 6.5	< 0.001	0.807	< 0.001	< 0.001
PSD [dB]	41	1.4 ± 0.9	1.2 ± 0.2	12.7 ± 2.5	< 0.001	0.936	< 0.001	< 0.001
pRNFL [µm]	41	98.4 ± 13.8	85.1 ± 11.9	63.6 ± 15.9	< 0.001	0.041	< 0.001	0.003
Macula [µm]	41	290.2 ± 9.6	281.0 ± 14.4	268.2 ± 12.4	< 0.001	0.113	< 0.001	0.059

*N* number of participants and selected eyes; except for age and sex, analysis was across all eyes rather participants for perimetric GLA group. *m* male; *f* female; *BCVA* best corrected visual acuity; *MD* mean deviation; *dB* Decibels for visual field deviation of 10 − 2 SITA standard VF; *PSD* Pattern Standard Deviation. *pRNFL* peripapillary Retinal Nerve Fibre Layer Thickness. 1- controls; 2- Preperimetric glaucoma patients without VF-loss; 3- Glaucoma patients with VF-loss.

### Visual field testing

#### Standard automated perimetry (SAP)

To compare the performance of RC in detecting central visual field defects, we used the 10 − 2 SITA algorithm test of the Humphrey Field Analyser 3 (Carl Zeiss Meditec AG, Jena, Germany).

#### Rapid Campimetry (RC)

Test design and procedure.

**Fig. 1:**
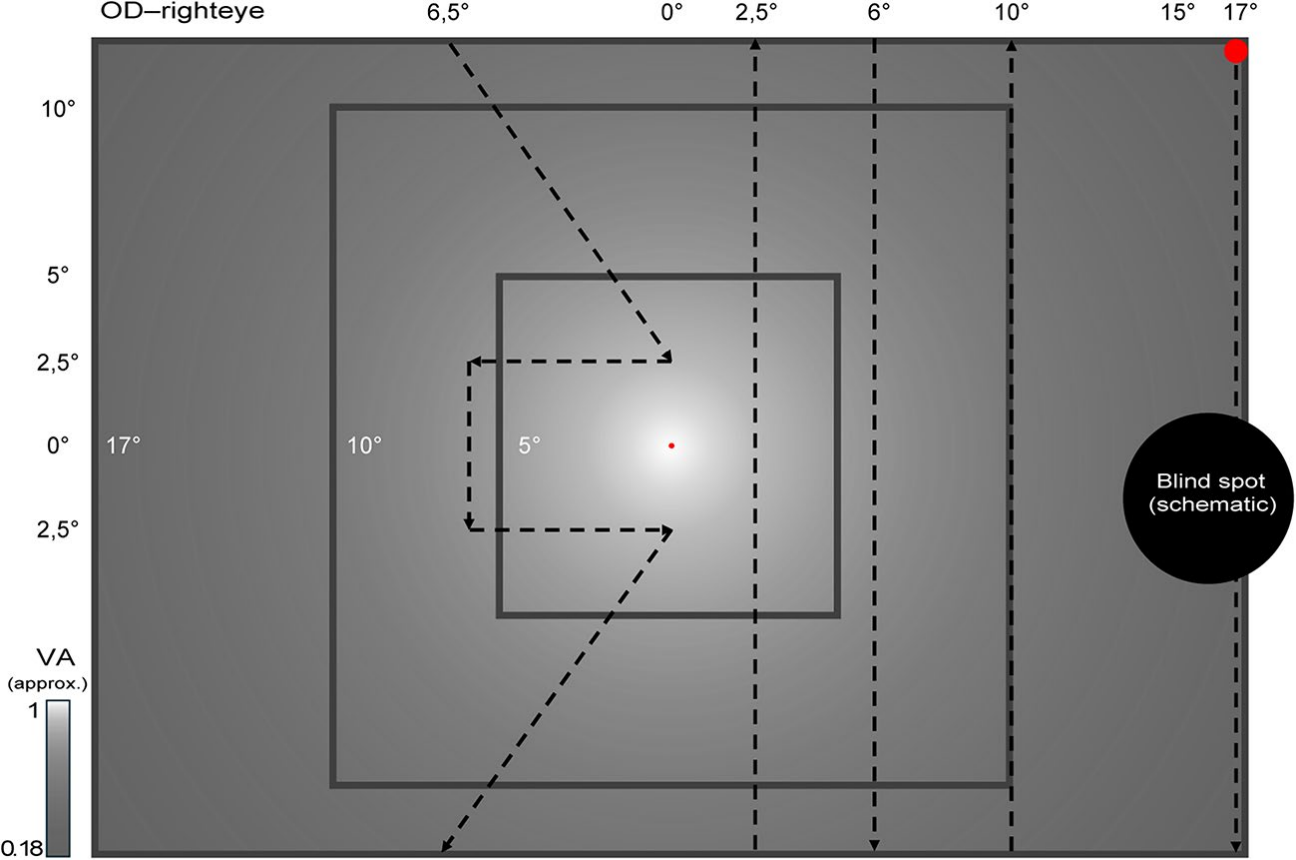
Rapid Campimetry path of the test point for the participant’s right eye. Participant sat 40 cm in front of the monitor. The dotted black line denotes the path travelled by the white suprathreshold stimulus. The testing always starts temporally, at the blindspot location. Test point changes its size as a function of visual acuity (VA) and cone density^22,23^ as indicated by the colorbar.

Rapid Campimetry, a kinetic-based VF test, is used to examine the visual field with detailed delineation of central defects. This test was performed before SAP tests with best corrected near vision in a dark room. An examiner controlled the stimulus and test on a separate monitor while a white suprathreshold stimulus moved across the VF at 40 cm on the investigation monitor as shown in Fig. 1. Participants had to fixate at a cross while responding to the moving stimuli (140 cd/m^2^) on a dark screen (0.8 cd/cm^2^). Close to the fixation target, the size of the test spot was 1.05 mm (0.16°), which automatically increased by 0.11 mm per degree and was 2.72 mm (0.39°) in the blind spot area. For this study, the VF test was extended to 17° horizontally to increase the chance of finding the blind spot and to ensure that the participants had understood the principle of the test, i.e., disappearance of test point at the scotoma areas. The examiner first performed the RC for screening, a short duration visual field test. When a VF loss was detected the Scotoma, RC in delineation mode was conducted to precisely delineate the scotoma (duration: 1–10 min). To reduce the possible loss of fixation, the patient was constantly reminded verbally to fixate during the test, see Müller et al.^18^ for further details. The examiner was blind to the 10 − 2 VF findings.

### Structural retinal examination

#### Optical coherence tomography (OCT)

##### OCT-acquisition

**Fig. 2:**
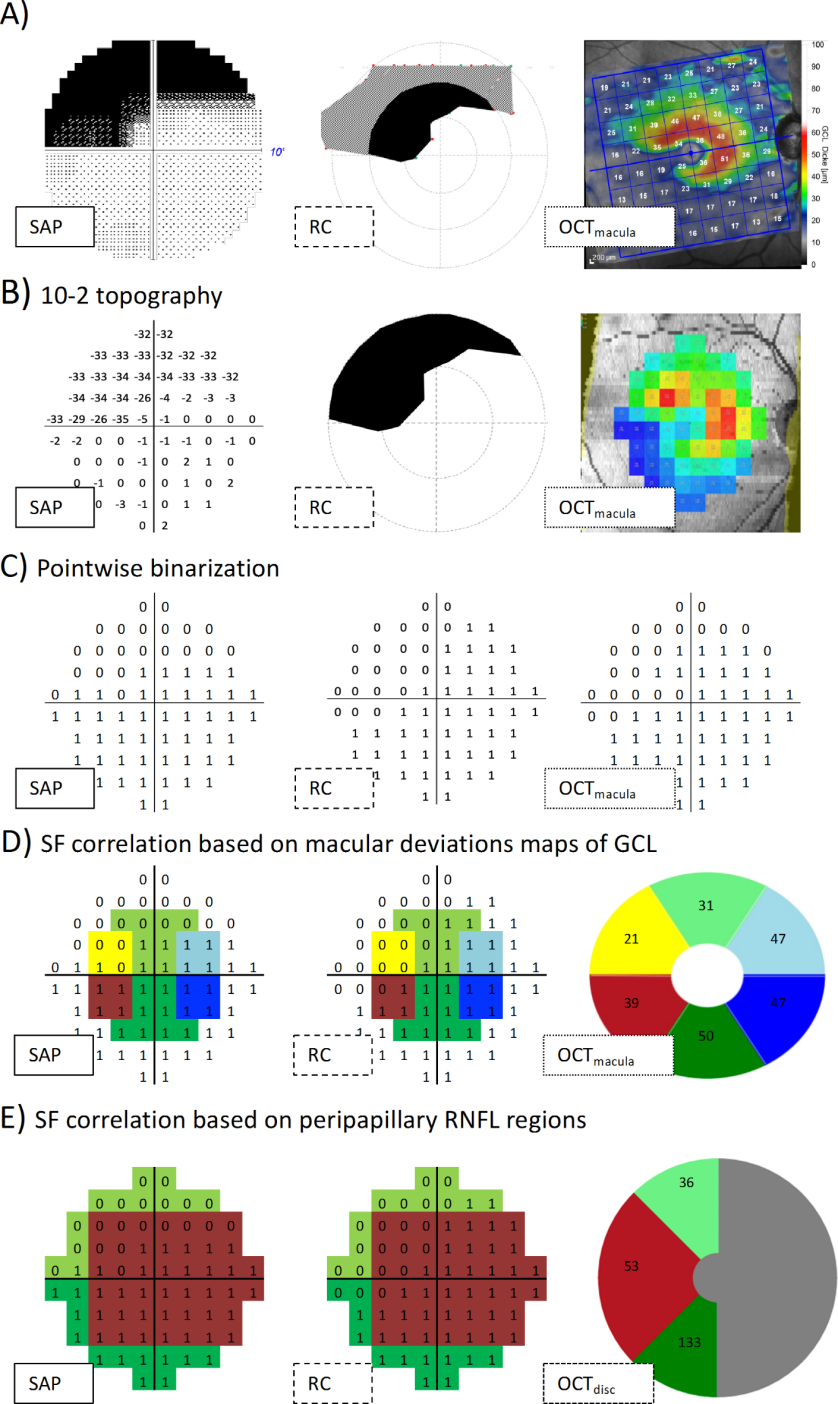
Correspondences maps for SAP, RC and OCT in a GLA. **A**) Original layout of each modality, 10-2 SAP, 17 x 10° RC and 30 x 25° OCT_macula_. **B**) Pre-processing of each method: 10-2 SAP mean deviations, 10 x 10° RC and 10-2 macular ganglion cell thickness using IOWA OCT analyzer. **C**) Extrapolation and binarization of responses where each point of 10-2 layout is coded as 0 (absolute scotoma) and 1 (no scotoma). SAP threshold to define absolute scotoma was MD≤ -30 dB; OCT_macula_ -based threshold was GCL thickness ≤ 20µm. **D**) Structure-function (SF) correspondence based on macular deviation maps regions accounting for foveal GCL displacement excluding the central 1.2 x 1 mm ellipse and 6 sector ellipse of 4.8 x 4 mm. **E**) SF correspondence based on with the respective regions at the peripapillary RNFL thickness area, namely superior temporal, temporal and inferior temporal; shaded grey area of the pRNFL is not included in the analysis.  GCL/pRNFL thickness topographies were flipped across horizontal meridian to match SAP field view.

We performed an OCT scan of the macula and optic disc to obtain a structure-function correspondence between RC and SAP vs. OCT. For OCT scans, we used the Spectralis glaucoma module (spectral-domain OCT; SD-OCT, ) software version 6.12.4 from Heidelberg Engineering, Germany. Glaucoma scanning module allowed scanning of the optic disc with a diameter of about 12 degrees and a horizontal B-scan of macula covering a 30 × 25-degree area of the retina, centred on the fovea. For one patient, pRNFL thickness was acquired 12 months later.

(A) Macula ganglion cell thickness (OCT_macula_):

(i) Macular volume scans were exported for further analysis with the Iowa Reference Algorithm (version 3.8.0, Retinal Image Analysis Lab, Iowa Institute for Biomedical Imaging, Iowa City, IA) to extract GCL maps within 10° with a 10 − 2 VF topography, i.e., 68 points with a 2° spacing. The OCT results are projected into the visual field for visualization and comparison with the visual field tests (Fig. 2A-C).
(ii) Accounting for histological displacement of retinal ganglion cells in the macula^24^, we also used Spectralis ellipse GCL thickness maps^25^. For this purpose, macular GCL thickness deviation maps were extracted from six wedge sectors in a 4.8 × 4 mm ellipse excluding the central 1.2 × 1 mm ellipse, which were related to their corresponding visual field parts as color-coded in Fig. 2D. OCT_macula_ maps are depicted in a top-down flipped manner to match VF with visual field view.

(B) Peripapillary retinal nerve fiber layer thickness (OCT_disc_, pRNFL): 

- pRNFL within a 3.5 diameter of optic disc were extracted for further analysis. pRNFL thickness for temporal, superior temporal and inferior temporal sectors were correlated with the corresponding estimates as suggested by Garway et al.^26,27^, see Fig. 2E. OCT_disc_ maps (Fig. 7), are depicted in a top-down flipped manner and as if obtained for the left eye.

### General analysis

#### Classifying VF defects

- VF defect: a contiguous of 3 abnormal hemifield points (*p* < 5%, 5% and 1% or 5%, 2% and 2%) in the central 10° VF on either total or pattern deviation maps. All VF defects are reliable if fixations loss and false negative rates < 33% and false positive rate < 20%.
- Arcuate scotoma: a dense scotoma that follows an arc and involves both quadrants.
- Relative arcuate scotoma: a relative (less dense) arcuate scotoma.
- Superior/Inferior hemifield VF defects: absolute defects involve both nasal and temporal quadrants.
- widespread: if the VF defect (relative/absolute) involves all 4 quadrants and not resembling arcuate scotomas.
- Others: Abnormal VF don’t belong to the above categories.

#### Modality correspondence with RC

RC tests was interpolated and extrapolated to depict a 10 − 2 VF layout for comparison with other methods. Since RC detects absolute VF defects, scotomas, and a cut-off value was determined for SAP and OCT to facilitate comparison across methods. For SAP 10 − 2, a mean deviation of any point ≤ -30 [dB] was considered a non-responsive location following Hood et al.^28^.

For structural OCT_macula_ data, inner retinal layer thickness reaches the floor for a SAP-MD of -10 dB^28–30^. This corresponded to macular GCL thickness of 20 μm^29^, a cut-off we employed for absolute scotoma designation of any point of 10 − 2 OCT topography. For OCT_disc_, peripapillary retinal nerve fiber layer thickness at the temporal region floored at 36–40 μm^31–33^ while other regions or average pRNFL floored at 43–49 μm^28,32,34,35^. As a priori, we sat 40 μm as floor level for temporal pRNFL thickness and 45 μm for other sectors.

The 68 corresponding points in the 10 − 2 map patters of each modality are binarized as detailed above into a response and non-response areas for further correspondence analysis. The correspondence analysis between tests were done only for those with VF defects demonstrable on the standard glaucoma test, SAP. The outcome estimates of correspondence were accuracy, sensitivity, and specificity calculated as follows^36^:

$$\mathrm{Accuracy}={\mathrm n}_{\mathrm{matched}\;\mathrm{locations}}/{\mathrm n}_{\mathrm{total}}$$

$$\mathrm{Sensitivity}\;=\;{\mathrm n}_{\mathrm{matched}\;\mathrm{abnormal}\;\mathrm{locations}}/{\mathrm n}_{\mathrm{abnormal}\;\mathrm{locations}\;\mathrm{in}\;\mathrm{reference}\;\mathrm{test}}$$

$$\mathrm{Specificity}\;=\;{\mathrm n}_{\mathrm{matched}\;\mathrm{normal}\;\mathrm{locations}}/{\mathrm n}_{\mathrm{normal}\;\mathrm{locations}\;\mathrm{in}\;\mathrm{reference}\;\mathrm{test}}$$

#### Effect size

Cohen’s kappa statistic ($\:\kappa\:$) is used to estimate the effect size of agreement levels between methods^37^. After chance agreement calculations, kappa statistic is calculated as follows^38^:

$$\:\frac{P\left(a\right)-P\left(e\right)}{1-P\left(e\right)}$$

Where P(a) is the actual observed agreement and P(e) is the chance agreement level. Interpretations of $\:\kappa\:$appa was following Landis and Koch criteria: 0 no agreement, ≤ 0.20 slight agreement, ≤ 0.4 fair agreement. ≤ 0.6 moderate agreement, ≤ 0.8 substantial agreement and > 0.8 almost perfect agreement.

## Results

**Fig. 3:**
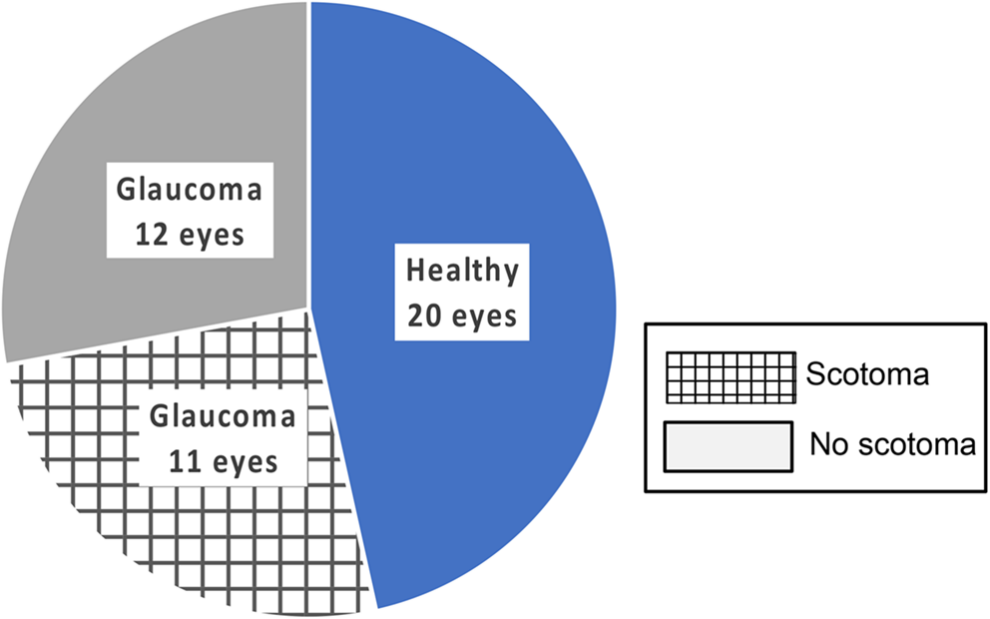
Detection of the presence/absence of a VF defect. Performance was identical for RC and SAP. VF defects were absent in 20/20 healthy participants (20/20 eyes) and 12/21 GLA (12/23 eyes) and present in 9/21 GLA (11/23 eyes).

Figure 3 summarizes the overall detection rate of glaucomatous VF defects with RC and SAP. Comparison RC and SAP – overview. Of the 32 eyes without VF defects (32 participants: 12 GLA, 20 HC), RC and SAP showed 100% agreement for the exclusion of any VF defects ($\:\kappa\:appa=1$). In turn, for the 11 eyes with VF defects (9 GLA), RC and SAP showed again 100% agreement for the detection of VF defects. In conclusion, the performance in the detection of the presence or absence of a VF defect was identical for RC and SAP.

### Comparison RC and SAP – individual VF grey plots

**Fig. 4:**
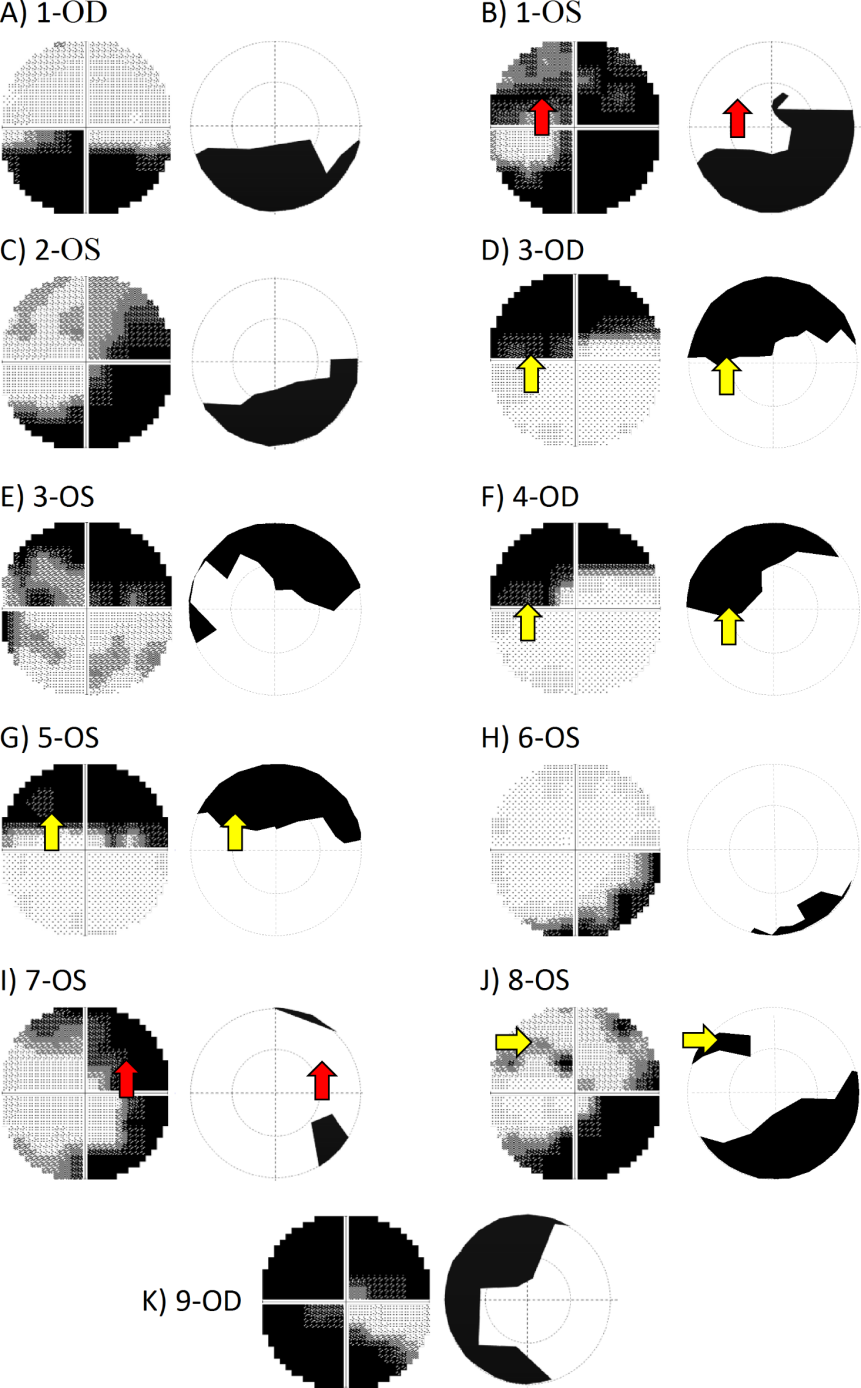
A-K) Patterns of VF defects on 10 − 2 SAP and RC grayscale plots. Both SAP and RC showed comparable scotomas in 5 eyes, namely A (VF hemifield loss), C, E and H (arcuate scotoma) and K (widespread VF loss). The other 6 eyes showed disagreement in showing arcuate scotoma as shown by red arrows (SAP better performance [B and I]) vs. yellow arrows (RC better performance [D, F, G and J]).

**Fig. 5:**
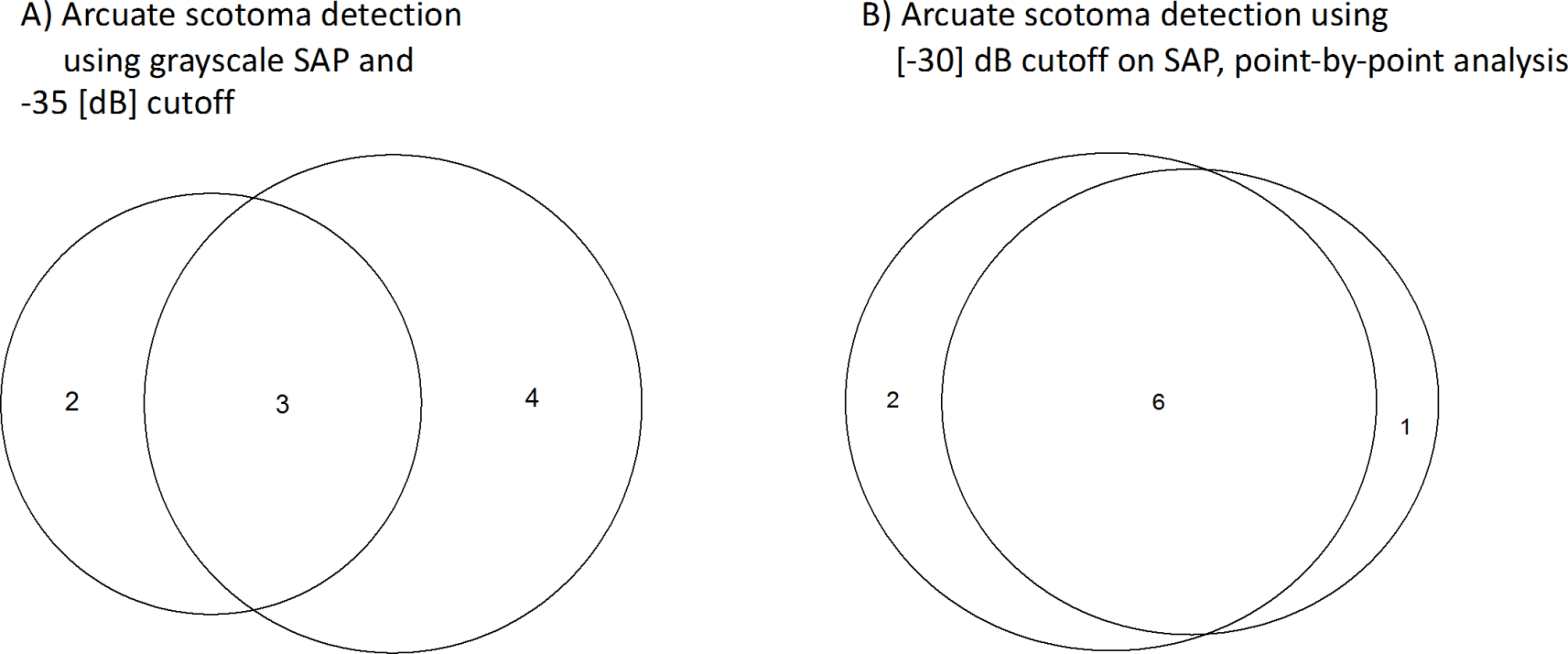
Absolute arcuate scotoma detection for SAP vs. RC given in Euler diagrams. (**A**) with a cut-off for absolute scotomas in SAP of -35 dB (SAP-RC agreement: 33%), (**B**) with a more liberal cut-off of -30 dB (SAP-RC agreement: 67%).

The individual VF plots of all 11 eyes with visual field defects are given in Fig. 4 as greyscale plots that depict the results from SAP 10 − 2 and RC. Due to the importance of topographical analysis of VF defects, rather than relying on the summary mean statistics, we compared the pattern of VF defects across methods at the individual level. Upon visual inspection of the VF topographies, both methods showed 5 comparable VF defects, namely a lower hemifield VF loss (Fig. 4A), arcuate scotomas (Fig. 4C, E and H), and a widespread VF loss (Fig. 4K). Since arcuate scotomas are more characteristic in glaucoma, we thus inspected its detection more specifically. Both methods showed a discrepancy in the detection of absolute arcuate scotomas in the other VFs, namely 2 on SAP (Fig. 4B and I) and 4 on RC (Fig. 4D, F, G and J). Figure 5 shows the Euler diagrams for absolute arcuate scotoma detections for the 9 GLA eyes with VF defects for two cut-offs for scotoma detection, i.e., cut-off of -35 dB (Fig. 5A) and a more liberal cut-off of -30 dB (Fig. 5B). SAP-RC agreement was 33% ($\:\kappa\:=0.32$) and 67% ($\:\kappa\:=0.74$), respectively.

### Quantitative comparison RC, SAP, OCT_macula_

**Fig. 6:**
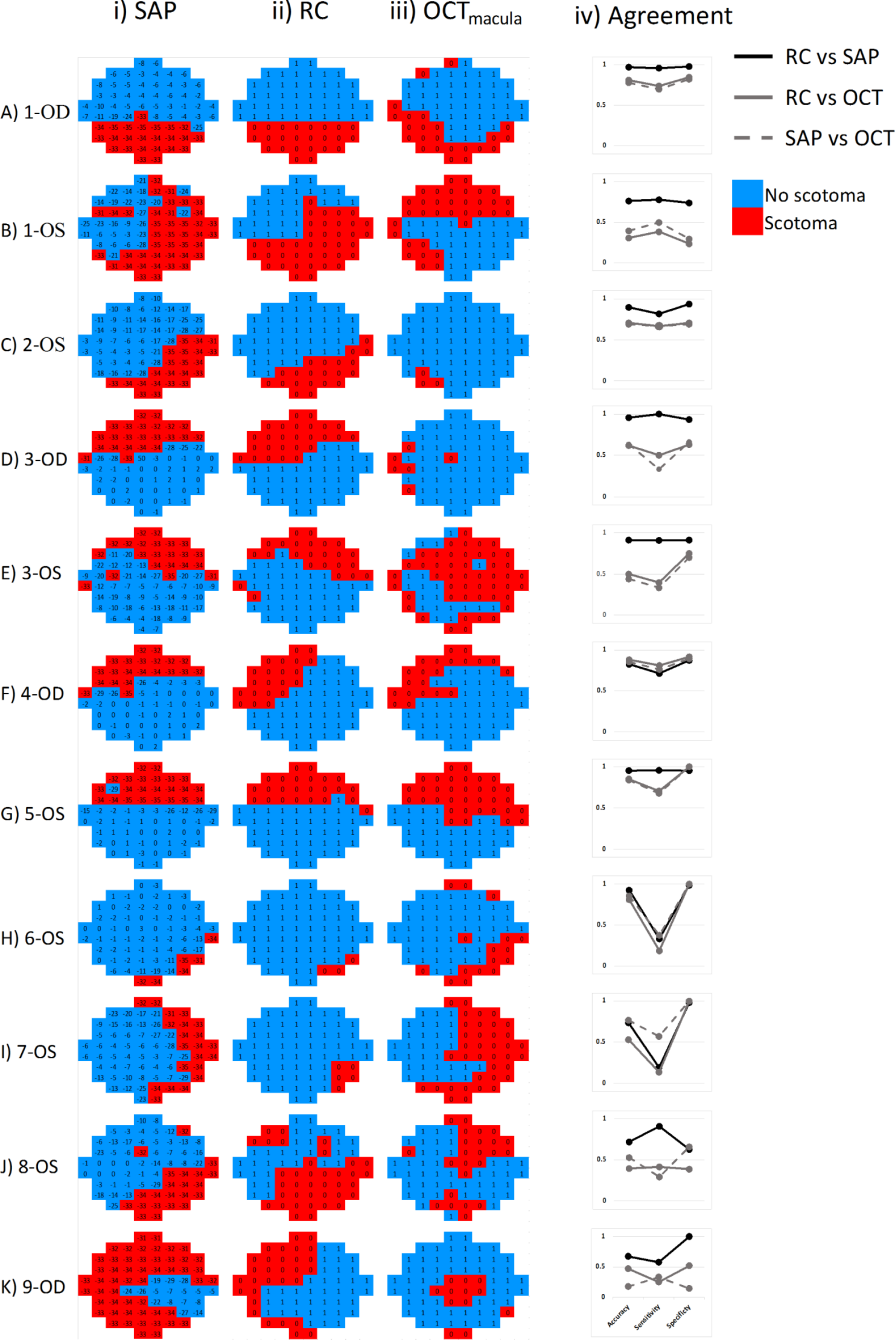
Pointwise macular structure-functional correspondence. (i) SAP, (ii) RC (iii) OCT_macula_, and (iv) cross-modal agreement as determined from accuracy, sensitivity and specificity. Data for the 11 eyes (9 participants) with absolute VF defects entered the analysis. Red/blue denotes presence/absence of an absolute scotoma. OCT_macula_ maps are flipped across the horizontal meridian to show the field view similar to the SAP and RC.

Next, we obtained a quantitative measure for the correspondence of the results from SAP, OCT_macula_ and RC. For this purpose, pointwise estimates of SAP and OCT_macula_ were binarized to response vs. non-response, as depicted in Figs. 2 and 6. Pointwise RC-SAP correspondence was highest for 7 GLA [Fig. 6A & C-H, columns (i) and (ii)] with agreement accuracy > 80%, see Fig. 6, panel iv. Moderate agreement (68–78%) was observed for the remaining 4 GLA [Fig. 6B, I-K, columns (i) & (ii)]. The overall RC-SAP correspondence showed an accuracy of 90% ($\:\kappa\:=0.77$ substantial agreement) with a median of 61 matched points out of 68 points. This corresponds to 82% sensitivity and 93% specificity. Structure-function (SF) pointwise correspondence was worse than reported above for the functional measures [Fig. 6 columns VF (i) and RC (ii) vs. OCT_macula_ (iii)]. The correspondence between the topographies of RC-OCT_macula_ [Fig. 6 column (ii) vs. (iii)] was highest for 4 GLA [Fig. 6A, F-H, columns (ii) & (iii)] with > 80% agreement accuracy, moderate, i.e., 62–70%, for 2 GLA [Figure C & D, columns (ii) & (iii)], and at or below chance level for 5 GLA [Fig. 6B, E and I-K, columns (ii) & (iii)]. The overall correspondence between the topographies of RC-OCT_macula_ reached a median accuracy of 62% ($\:\kappa\:appa=0.10;\:$42/68 matched points), 50% sensitivity, and 75% specificity. For comparative purposes, we also determined SAP-OCT_macula_ correspondence [Fig. 6 columns (i) & (iii)], which was comparable to RC-OCT_macula_ with a 69% median accuracy ($\:\kappa\:appa=0.33$; 47/68 matched points), 50% sensitivity, and 70% specificity. Here, we observed highest agreement (> 80%) for 3 GLA [Fig. 6F-H, columns (i) & (iii)], moderate (63–78%) agreement in 4 GLA [Fig. 6A, C, D & I, columns (i) & (iii)] and at or below chance level for 4 GLA [Fig. 6B, E, J &K, columns (i) & (iii)].

**Fig. 7:**
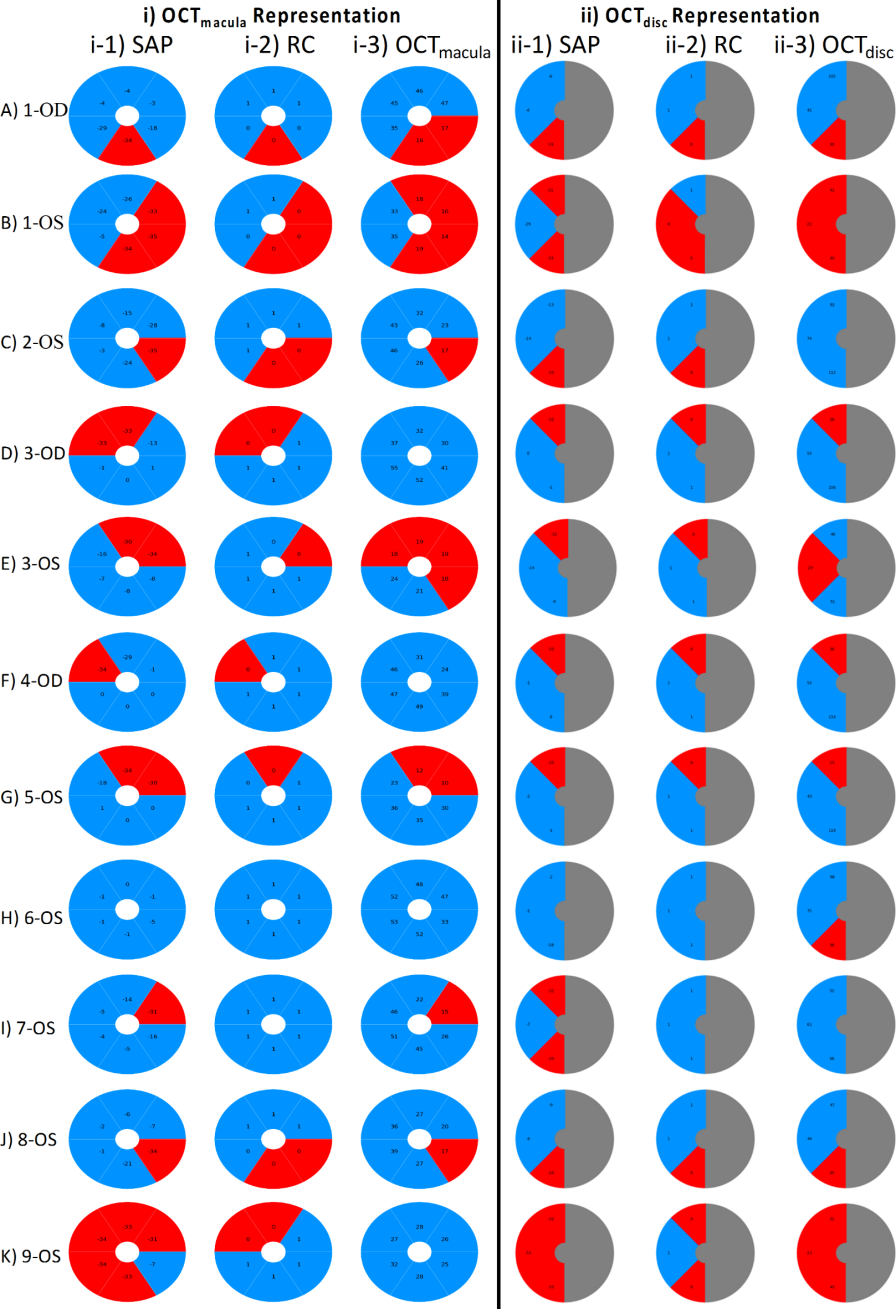
VF locations sectorial grouping and structure-function (SF) correspondence with macula and disc OCT. i) Macula-related deviation-map construction of SAP (1), RC (2) and OCT_macula_ (3) that account for GCL displacement using a 6-sector ellipse of 4.8 × 4 mm excluding the central 1.2 × 1 mm of all modalities. ii) Optic disc-related map construction of SAP (1), RC (2), and OCT_disc_ (corresponding to the 3 sectors of optic disc, namely temporal, supertemporal & inferotemporal sectors. For illustrative purposes, we show the grouped SAP and RC locations as per the corresponding sector of the pRNFL thickness. The nasal part of OCT_disc_ (grey) is masked out as it has no direct relation to VF. The nasal part of SAP and RC maps is masked out for presentation purposes and remained part of the analysis as detailed in Fig. 2. The SAP, RC and OCT_disc_ maps are depicted as the field view of left eye. Red/blue denotes absolute/no scotoma.

Employing the above approach of pointwise correspondence between modalities benefited from the high spatial resolution of OCT_macula_ at the expense of accounting of retinal ganglion cell displacement at the fovea. We repeated the analysis to incorporate the latter at the expense of spatial resolution and adopted sector analysis for SF relationship at the macula following previous reports^24,25^. Here, we grouped VF-deviation locations using the median value to match the corresponding sector of the macula GCL thickness, OCT_macula,_ [see Figs. 2D and 7 columns (i-1) SAP, (i-2) RC & (i-3) OCT_macula_]. For each eye, the OCT_macula_ maps are flipped in horizontal meridian to match the corresponding VF view and we excluded points outside the specified ellipse, see methods for further details. Further we report all data obtained for the 7 OS and 4 OD eyes. This sector analysis approach resulted in better SF correspondence where RC-OCT_macula_ agreement, [Fig. 7 columns (i-2) & (i-3)], reached an overall accuracy of 83% with 50% sensitivity and 100% specificity, ($\:\kappa\:appa=0.57\:\left[56\right],\:moderate\:agreement$). Here, 7 vs. 4 GLA had SF correspondence of 80–100% [Fig. 7A-C & F-I, columns (i-2) and (i-3)] vs. 50–67% [Fig. 7D, E, J &K, columns (i-2) & (i-3)]. SAP-OCT_macula_ correspondence, [Fig. 7 columns (i-1) & (i-3)], was likewise better with an overall accuracy 83% accuracy with 100% sensitivity and 100% specificity, ($\:\kappa\:appa=0.57\:\left[56\right],\:moderate\:agreement$). Here, 7 GLA had an accuracy of 83–100% [Fig. 7A-C & G-J columns (i-1) and (1–3)], 3 GLA had an accuracy of 67% [Fig. 7D-F, columns (i-1) & (i-3)]) and in one GLA accuracy was 16% [Fig. 7k, columns (i-1) & (i-3)]. Upon the use of IOWA OCT_macula_, as a sanity check, showing ellipse macular maps, SAP and RC vs. OCT_macula_ correspondence improved to 83% and 67%, respectively. Segmentation algorithms differences for retinal layers between Heidelberg and IOWA might account for such differences.

### Quantitative comparison RC, SAP, OCT_disc_

We also discerned SF relationship using the OCT peripapillary retinal fiber layer (pRNFL) thickness estimates, OCT_disc_, and we grouped VF locations according to the corresponding pRNFL sector^26^, namely the temporal, superior temporal and inferior temporal sectors, see Figs. 2E and 7 column (ii). VF locations are grouped as per corresponding pRNFL sectors for both SAP [Fig. 7 column (ii-1)] and RC [Fig. 7 column (ii-2)]. Here, VF maps of the 3 modalities are shown as the field view of left eye with the nasal part of the VF maps masked out. Here, all locations of SAP and RC are included in the analysis, but the nasal mask is shown for illustrative purposes. RC was in perfect agreement with OCT_disc_ median estimates of 100% accuracy, 100% sensitivity, and 100% specificity, respectively, ($\:\kappa\:appa=1$). For RC-OCT_disc_ correspondence, 6, 4 and 1 GLA had an accuracy of 100% [Fig. 7A, D, F, G, I & J, columns (ii-2) & (ii-3), 67% [Fig. 7B, C, H & K, columns (ii-2) & (ii-3)] and 33% [Fig. 7E, columns (ii-2) & (ii-3)], respectively. SAP-OCT_disc_ correspondence was also in perfect agreement with a median estimate of 100% accuracy, 100% sensitivity, and 100% specificity, respectively, ($\:\kappa\:appa=1$). Here, 6, 3 and 2 GLA had an accuracy of 100% [Fig. 7A, D, F, G, J & K, columns (ii-1) & (ii-3)], 67% [Fig. 7B, C and H, columns (ii-1) & (ii-3)] and 33% [Fig. 7E & I, columns (ii-1) & (ii-3)], respectively. For each eye, the OCT_macula_ and OCT_disc_ maps are flipped across horizontal meridian to match the corresponding VF view and we excluded points outside the specified ellipse, see methods for further details. Further we report all data for OCT_disc_ as if obtained for the left eye.

## Discussion

### Summary of findings

This study investigated the correspondence of Rapid Campimetry (RC) with 10 − 2 standard automated perimetry and OCT_macula_ macular ganglion cell layer thickness (GCL) and OCT_disc_ peripapillary retinal nerve fiber layer thickness (pRNFL). For VF defects detection, both RC and SAP showed 100% agreement for the exclusion of VF defects in HC and preperimetric glaucoma as well as 100% agreement for the presence of VF defects in glaucomatous eyes, 11 of 9 participants. Both RC-SAP performed comparably in the detection of different scotoma patterns. Pointwise correspondence, RC-SAP agreement reached 90% accuracy; for structure-function (SF) correspondence, RC [SAP] pointwise agreement with GCL, OCT_macula_, was 62% [69%]. Accounting for GCL displacement at fovea, RC [SAP] agreement with OCT_macula_ reached 83% [83%]. Finally, RC [SAP] agreement with pRNFL, OCT_disc_, was highest with a median accuracy of 100% [100%].

### Kinetic-based vs. static visual field testing

The utility of kinetic visual field (VF) testing in glaucoma diagnosis might hold high potential in filling gaps in SAP testing, namely high test-retest variability and limited dynamic range in advanced glaucoma cases. Recently, this issue received more attention. Two techniques applied kinetic-based VF testing of stimulus crossing/following the trajectories of retinal nerve fiber cells^18,39^. Rapid Campimetry promise high potential in glaucoma VF tests where it was shown that kinetic VF testing has lower test-retest variability^19^. Further, Kinetic perimeter showed higher dynamic range than SAP testing^39^. We have also shown that the RC has high test-retest reproducibility and being robust to common suboptimal environment testing^19^. However, our setup with RC, which needs only a computer and a screen, is more compatible for telemedical VF testing in remote areas and even at home than the conventional perimetry setup of Gardiner et al.^39^. Moorfield motion displacement test, a task to determine the smallest perceptible positional displacement leading to the sensation of motion, is another psychophysical motion perimeter test with sensitivity and specificity of 80–85% to detect glaucoma damage outperforming SAP at early glaucoma detection^40^ and Invest Ophthalmol Vis Sci 49:ARVO E-abstract 4080, 2008]. However, for such tests to be incorporated in clinical practice, further quantifications in comparison to SAP and/or OCT are warranted.

### RC vs. SAP and OCT correlations

In general, motion-like or kinetic-based VF tests showed consistent results with the conventional SAP test in glaucoma as shown by previous studies^41^(p19). Beck et al. reported, in line with our findings, an 88% agreement between SAP and Goldmann kinetic perimeter in glaucomatous and neurologic cases. A following study using motion coherent perimetry, recognition of a position shift of a dot against fixed dots background, reported superior performance of this type of perimetry in comparison to SAP in early glaucoma cases but with significant correlations of the probability plot analysis of both techniques^42^. Another recent VF test, Vivid Vision Perimetry (VVP; Vivid Vision, Inc) employing oculo-kinetic perimetry, where the patient moves his/her eye to visualize the test target, showed very significant agreement, in line with our findings, (0.86 and 0.63 Pearson correlation) between VVP vs. SAP and OCT_macula_^43^.

In the present study, Rapid Campimetry has been correlated systemically to standard tests in glaucoma namely SAP and OCT, a novel attempt not pursued by the other novel kinetic visual field tests. The pointwise approach helped to prove that RC identifies localized damage in comparison to SAP and OCT_macula_. Further, accounting for foveal displacement for retinal ganglion cells is another informative approach demonstrating better SF relationship than pointwise correspondence. OCT_disc_ (RNFL thickness) agreed better with RC/SAP than OCT_macula_ (GCL thickness) in most glaucoma patients with moderate VF loss. These results are also consistent with previous studies- investigating SF relationship between conventional glaucoma measures (SAP and OCT)- that found a more linear relationship between RNFL and VF in early and moderate glaucoma, and a non-linear relationship with GCL thickness in late severe glaucoma, where even small changes in GCL thickness led to large changes in VF^44^. In contrast to our results, Tong et al. and Hirasawa^45,46^ found no improvement by accounting for the fiber shift at the fovea for SF comparisons. However, when reviewing the Drasdo method and the Tong et al. publication, Montesano et al.^47^ also found a better SF correlation accounting for the displacement with better agreement when VF defects within 20 degrees.

In any case, RC proved to have reproducible tests to standard tests, more importantly to functional SAP, standard VF test in glaucoma management, and this promises high potential for future implementations in clinics and telemedical tools given its features: (i) suprathreshold stimulus, (ii) plausibility of VF defects that complement SAP tests, (iii) fast exam test in < 1 min in screening mode, and (iv) easily mounting test setup.

### Limitations, future perspectives

We acknowledge few limitations of the Rapid Campimetry approach as applied in the present study. One limitation is a small size of participants which ought to be validated in larger and multicentric cohorts. Further, RC can only detect absolute VF defects and it does not take into account relative scotomas. It should be noted though that we have shown that RC can detect absolute scotomas that are appear relative in SAP. Another limitation is the absence of fixation monitoring and assessment. Here, we performed a check on fixation control similar to Heijl’s methods, where the test started with the projection of the test point into the blind spot area^48^ by extending RC test area to 17° horizontally. Detection of the blind spot was considered as a confirmation of fixation by the patient. During the test, the patient was also observed by the examiner and reminded to maintain fixation. Another important factor contributing to fixation stability of test is the short duration of the test which keep patient more in focus and less bored. Acknowledging the importance of testing of VF outside 10°, albeit the given the evidence of this region importance both in early and late glaucoma, future development of RC will extend tested VF to 30°. Finally, further optimization to the tests in a multicenter study will contribute to establishing RC as a valuable complementary VF test in glaucoma.

## Conclusion

In summary, Rapid Campimetry, a new kinetic VF test, gave reproducible results similar to standard VF testing, SAP, with high accuracy and substantial agreement. The perfect agreement to detect/exclude VF defects stimulate its utility as a screening VF test given its very short test duration, < 1 min in screening mode, and its compatibility with cloud technologies. Further optimization of the RC-test, specifically automation and extension of tested VF to periphery, in a future multicenter study will lay the foundations of establishing a new telemedical fast functional kinetic campimetry in glaucoma practice.

## Electronic supplementary material

Below is the link to the electronic supplementary material.

Supplementary Material 1

## Data Availability

Data available upon request. Please contact the corresponding author, MBH, for data requests.
