# Supplementary material for: Rapid Campimetry in glaucoma – correspondence with standard perimetry and OCT
Source: Sci Rep. 2024 Oct 25;14:25400. doi: 10.1038/s41598-024-75037-5 (PMC11511816; doi:10.1038/s41598-024-75037-5)
Supplement: Supplementary file 1 — Supplementary Material 1 [file 41598_2024_75037_MOESM1_ESM.pdf]

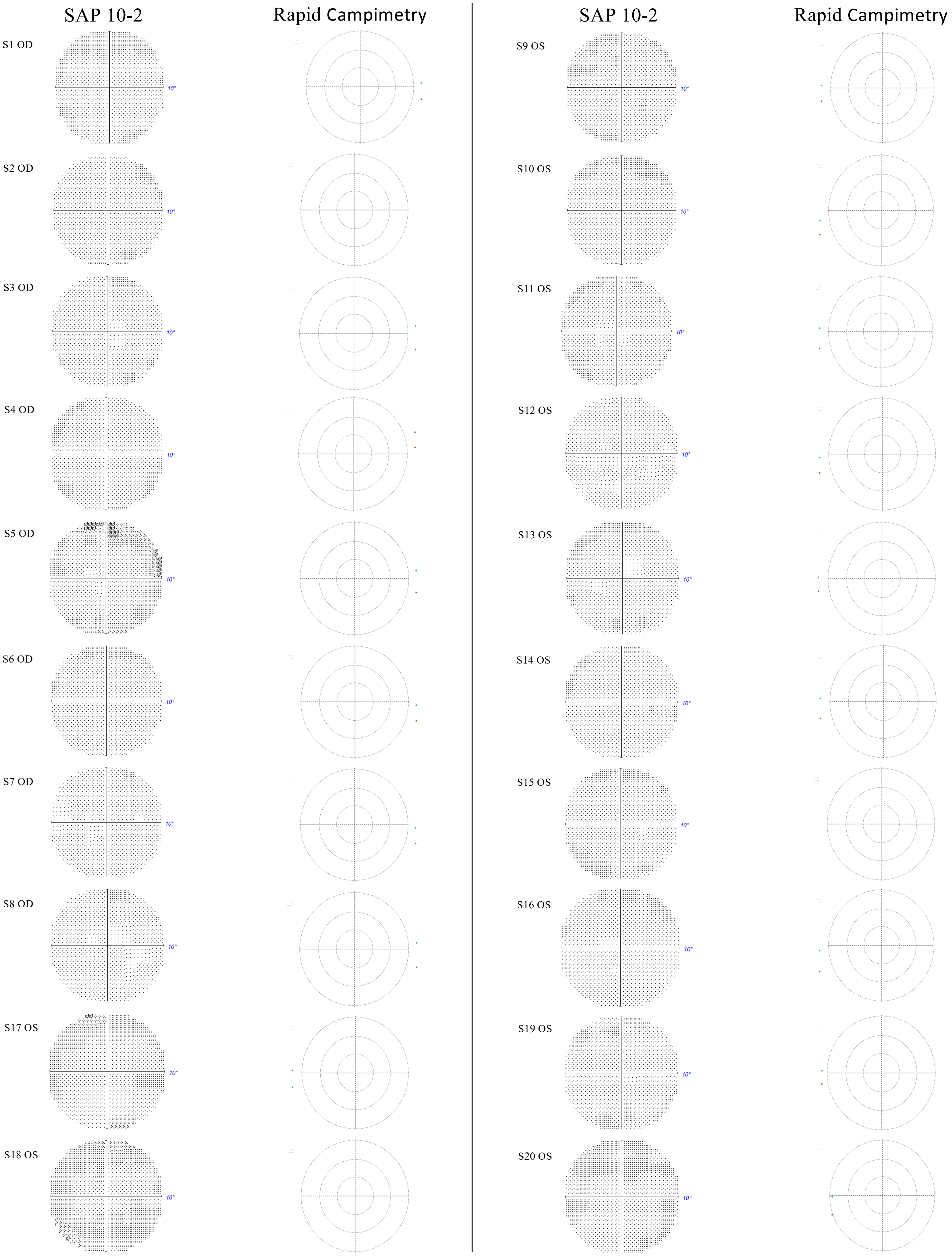

Supplementary Figure 1. Grayscales of standard automated perimetry (10° VF) and Rapid Campimetry in 20 HC Eyes (17°x10° VF). Red and green dots in RC delineates the blindspot boundaries.

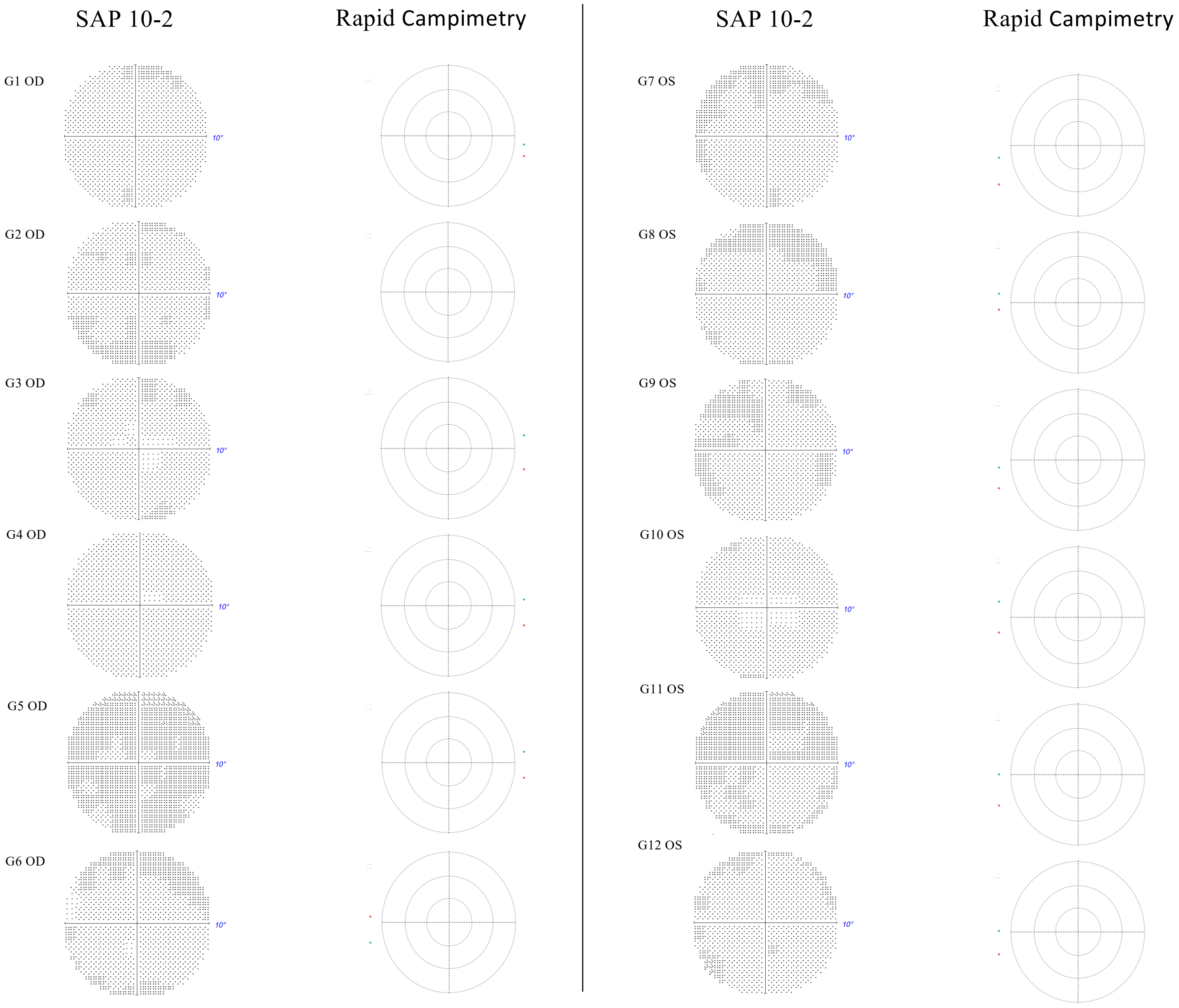

Supplementary Figure 2. Grayscales of standard automated perimetry (10° VF) and Rapid Campimetry in 12 preperimetric glaucomatous Eyes (17°x10° VF). Red and green dots in RC delineates the blindspot boundaries.
